# Right Forceps Minor and Anterior Thalamic Radiation Predict Executive Function Skills in Young Bilingual Adults

**DOI:** 10.3389/fpsyg.2018.00118

**Published:** 2018-02-09

**Authors:** Ping C. Mamiya, Todd L. Richards, Patricia K. Kuhl

**Affiliations:** ^1^Institute for Learning and Brain Sciences, University of Washington, Seattle, WA, United States; ^2^Department of Radiology, University of Washington, Seattle, WA, United States

**Keywords:** Stroop task, reaction time, tract-based spatial statistics, tensor mode, fractional anisotropy, fasciculation

## Abstract

Executive function (EF) skills enhance learning across domains, and are particularly linked to the acquisition of a second language. Previous studies have shown that bilingual individuals show enhanced EF skills in cognitive tasks where they attended a targeted dimension of a stimulus while inhibiting other competing cues. Brain imaging revealed that bilingual young adults’ performances in the Stroop color-naming task were related to the volume of anterior cingulate cortex (ACC) and inferior frontal lobe. Subjects who had greater white-matter in the frontal cortex showed enhanced performances in the same task, suggesting that brain fiber pathways connecting ACC to the frontal region may be related to the Stroop color-naming task. No studies to date have examined the tissue properties of brain fiber pathways connecting these brain regions and their association with subjects’ EF performances. Importantly, there are no data establishing whether bilingual subjects exhibit different reaction times when words are presented in their first versus second language. To study these questions, we used behavioral and unbiased whole-brain analyses, recruiting 21 Chinese students. Using the Stroop color-naming task, we measured subjects’ reaction times (RTs) in which color names were displayed using fonts that matched the named color (congruent task) or mismatched the color (incongruent task). Students performed the task twice, first in English, the subjects’ second language, then in Chinese, the subjects’ primary language. Results from whole-brain analysis showed that students’ RTs in both the English and Chinese tasks were significantly correlated with the mode of anisotropy (MO) in a brain cluster containing the forceps minor and anterior thalamic radiation in the right hemisphere. We also found that fractional anisotropy (FA) significantly predicted students’ RTs, with higher FA predicting shorter RT. Taken together, our findings demonstrate that right forceps minor and anterior thalamic radiation predict EF skills, suggesting that this brain feature may be important for young bilingual adults using their first and second languages to direct their attention when conflicting cues are present.

## Introduction

Executive function (EF) skills are malleable. One skill, the ability to attend to a specific representation while inhibiting irrelevant cues, has been shown to be enhanced through bilingual experience (Bialystok et al., 2005, 2008) [for reviews, see (Bialystok et al., 2012; Costa and Sebastian-Galles, 2014)]. Current thinking is that bilingual individuals learn to select the intended language while inhibiting the use of the other language. This type of switching between two languages requires bilingual individuals’ EF skills. For example, in cognitive tasks where bilingual individuals were required to name objects, they showed longer times to respond when using their dominant language following a task where they named an object using their less dominant language. It is thought that the longer time required to respond using their dominant language may be related to persistent attention control carried over from the first task in which they were required to respond using their second or less proficient language (Costa and Santesteban, 2004; Branzi et al., 2016).

When competing representations are present simultaneously, bilingual individuals also show enhanced EF. For example, bilingual individuals showed enhanced performance in identifying the color of a printed font in the Stroop color-naming task (Bialystok et al., 2005; Costa et al., 2008; Martin-Rhee and Bialystok, 2008; Yang et al., 2011; Coderre and van Heuven, 2014). The Stroop color-naming task, introduced more than 80 years ago by Stroop (1935), has been commonly used to assess subjects’ attention control skills [for reviews, see (MacLeod and MacDonald, 2000; Grundy et al., 2017)]. Rather than using a habitual response when naming a printed word that names a color, subjects are required instead to direct their attention to the color of the printed font. It has been shown that subjects require a longer time to respond in the condition when the color mismatches the meaning of a printed font (incongruent), compared to the one when color and meaning match (congruent) (MacLeod and MacDonald, 2000). Taken together, findings from these behavioral studies demonstrate that bilingual individuals show enhancement in their EF skills by attending to a targeted dimension of a stimulus while suppressing competing cues in the same stimulus.

There is evidence that bilingual individuals’ responses in the Stroop color-naming task are related to volumes of brain gray- and white-matter structure. Takeuchi et al. (2012) presented English fonts to Japanese–English bilingual college students and found that students’ responses to the fonts printed in English, the second language of the students, were significantly correlated with the volumes of cingulate cortex and inferior frontal gyrus. The same study also showed that the students’ performances were positively correlated with the volume of white matter in the frontal lobe. These authors suggest that the observed relationship between brain white- and gray-matter volumes and the students’ performance in the task may be related to increased brain fiber pathways in the connected regions. However, no one to date has examined whether the properties of brain fiber pathways connecting cingulate cortex and frontal lobe are related to bilingual individuals’ performances in the Stroop color-naming task.

Structural properties of brain fiber pathways have been linked to cognitive functions, including second language learning in bilingual adults. Using the diffusion-tensor imaging (DTI) technique, bilingual individuals who have acquired a second language earlier in their life or have lived in a second language immersive environment for a substantial period of time have higher fractional anisotropy (FA) than their monolingual counterparts (Luk et al., 2011; Mohades et al., 2012, 2015; Pliatsikas et al., 2015). Interestingly, bilingual individuals who have acquired a second language relatively later in life or had a shorter length of immersive experience have lower FA compared to monolinguals (Cummine and Boliek, 2013; Gold et al., 2013; Kuhl et al., 2016).

Diffusion-tensor imaging has been increasingly used to describe the structural properties of brain fiber pathways *in vivo* at various ages [for example, (Tamnes et al., 2012); for a review, see (Zatorre et al., 2012)]. FA reported in the above studies is a measure of the net directionality of water diffusion, and indirectly linked to the physical properties of axonal fibers, including increased myelination, higher axonal branching, and reduced volume in the extracellular space around brain fiber pathways [for a review, see (Fields, 2008)]. Adults who showed higher FA in a brain fiber pathway connecting the frontal region to the posterior language area had better second language learning outcomes than the individuals who showed lower FA in the same brain fiber pathway (Schlegel et al., 2012; Qi et al., 2015; Mamiya et al., 2016).

Although FA measured in this brain fiber pathway is related to second language experience, it fluctuates in brain fiber pathways connecting distant brain regions. It is thought that fiber crossings in a different direction may have contributed to this variability (Jbabdi et al., 2010). A recent study by Douaud et al. (2011) combined FA and another DTI index, mode of anisotropy (MO), to show an increased MO and a co-localized FA in a region of crossing fiber in patients with mild cognitive impairment (Douaud et al., 2011). Similarly, patients with obsessive-impulsive disorder also show various FA values in several long-range fiber pathways that may be related to fiber crossing (Radua et al., 2014). When MO and FA values are approximately close to 1, fiber pathways in a given voxel are orientated in a predominant direction, which can result in a linear shape of diffusion tensor in the DTI^1^. On the other hand, a negative MO value and a reduced FA indicate that two fiber pathways may cross each other at a perpendicular direction. This will likely result in a planar shape of the diffusion tensor. The results from these studies suggest that taking into account crossing-fibers in a voxelwise analysis is important to understanding how the structural properties of brain fiber pathways are related to cognitive functions.

To understand whether the properties of brain fiber pathways are related to bilingual individuals’ performances in the Stroop color-naming task, we used the DTI technique and quantified the structural properties of brain fiber pathways using FA and MO. We hypothesized that brain fiber pathways connecting the anterior cingulate cortex and frontal lobe are related to performance in the Stroop color-naming task. Bilingual subjects with higher FA and MO are expected to have enhanced performance in the task when compared to individuals with lower FA and MO. We also explored whether students’ performances in the task differed depending on whether the fonts were printed in subjects’ dominant language as opposed to their second language.

To test this hypothesis, we used DTI and conducted an unbiased whole-brain analysis examining whether brain fiber pathways exist with tissue properties that predict bilingual subjects’ responses in the Stroop color-naming task. The task also allowed us to investigate whether students’ responses depend on the language used in the task.

We recruited Chinese college students who enrolled in the University of Washington as full-time students. We used the Stroop color-naming task and asked subjects to name the colors of fonts printed on the screen in two separate tasks (Pardo et al., 1990). In the first task, the fonts presented were printed in English, the second language of our subjects. In the second task, the same words were printed in Chinese, the first language of our subjects. We investigated: (a) whether there were brain regions in which the tissue properties of brain white matter correlated with students’ reaction times (RTs) in these tasks, (b) whether the relationship identified in (a) would differ between the tasks performed in the first versus the second language, and (c) whether subjects’ RT in the English Stroop task correlated with their RTs in the Chinese Stroop task.

## Materials and Methods

### Selection Criteria

All subjects were new full-time students enrolled at the University of Washington (*N*_male_ = 10, *N*_female_ = 11; mean age_male_ = 21, standard deviation_male_ = 3.559; mean age_female_ = 24, standard deviation_female_ = 4.646), and they were part of a cohort that was previously reported (Mamiya et al., 2016). All experimental procedures were approved by the Institute Review Board of the University of Washington, and written informed consent was obtained from each participant. All students had similar English-learning histories. That is, all were exposed to English in schools in China. The mean age of acquisition was 9.9-years of age (*SE* = 0.07). We excluded subjects who had lived outside of China prior to moving to the United States; previously participated in a student-exchange program outside of China; had a mother or father who is not of Chinese origin; used serotonin or dopamine-related agents in the past; had a medical history of Axis I disorders, epilepsy, or brain injury; had problems with vision or hearing; or were left-handed as assessed by the Edinburgh handedness test. For MRI safety, individuals with metallic or cardiac implants or tattoos were additionally excluded from the study.

### Color-Naming Version of the Stroop Task

**FIGURE 1:**
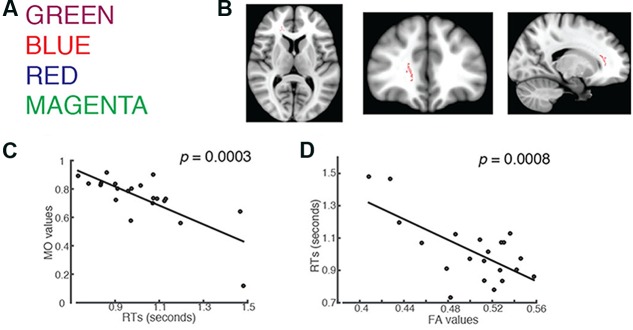
Students’ RTs were significantly negatively correlated with the MO values for: **(A)** words printed in a color that mismatches the color name (incongruent trials). **(B)** Axial, coronal and horizontal views of a brain cluster (shown in red) where the MO values were significantly negatively correlated with students’ RTs. MNI Coordinates of the peak voxel were: *X*: 18, *Y*: 35, *Z*: 9. The total number of voxels in the cluster was 137. **(C)** A negative relationship between students’ RTs in the incongruent trials and the MO values in the brain cluster shown in **(B)**. **(D)** FA values in the brain cluster shown in **(B)** were used in the linear regression model to predict students’ RTs recorded in the incongruent condition. Results from the linear regression analysis showed that FA significantly predicted subjects’ RTs and explained 45.29% of the total variances in students’ responses in the task (*R*^2^ = 0.4529, *p* = 0.0008).

The experiment was conducted using the MacBook Pro with Retina (15-inch) display. It had a screen resolution of 2880-by-1800 at 220 pixels per inch. We used the MATLAB script to randomly present one of the four words, BLUE, RED, GREEN, and MAGENTA (**Figure 1A**) on the monitor. Students were required to identify the color of the presented font by pressing the corresponding key on the keyboard. The corresponding key for red color was R, for green color was G, for magenta color was M, and for blue color was B. The presented font remained on the screen for 2 s unless a key was pressed. All subjects were instructed to respond as quickly as possible. An invalid trial was marked if there was no key press after the presentation of a font for 2 min. The computer recorded the latency between the time when a font was presented and the time when a key was pressed. This latency represented a subject’s RT in a single trial. All subjects had to make 100 correct responses in order to finish the task, and all subjects successfully completed the task.

**FIGURE 2:**
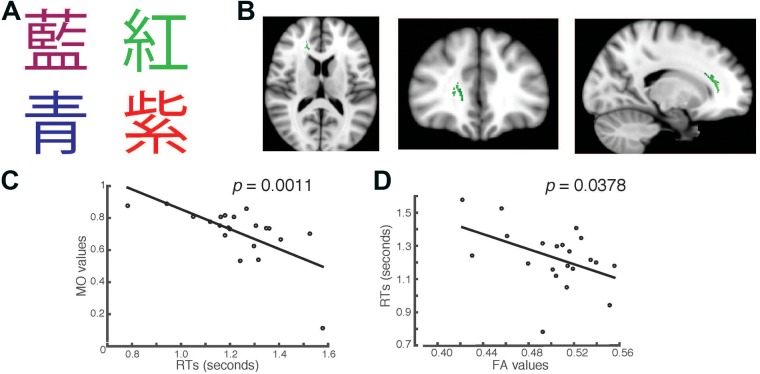
Students’ RTs were significantly negatively correlated with the MO values **(A)** when the Chinese fonts presented did not match the named color in incongruent trials. **(B)** Axial, coronal and horizontal views of a brain cluster (shown in green) where the MO values were significantly negatively correlated with students’ RTs. MNI Coordinates of the peak voxel were: *X*: 18, *Y*: 35, *Z*: 9. The total number of voxels in the cluster was 335. **(C)** A negative relationship between students’ RTs in the incongruent trials and the MO values in the brain cluster shown in **(B)**. **(D)** FA values of the brain cluster identified in **(B)** were used in a linear regression model to predict students’ RTs recorded in the incongruent condition. Results from the linear regression analysis showed that FA values significantly predicted students’ RTs and explained 20.78% of the total variance in students’ responses in the task (*R*^2^ = 0.2079, *p* = 0.0378).

After subjects completed the first task, we then presented the same words in a second task in which the words were printed in traditional Chinese characters (**Figure 2A**). As described in the first task, the corresponding key for red color shown in Chinese character was R, green color in Chinese character was G, magenta color in Chinese character was M, and blue color in Chinese character was B. There were two conditions in both English and Chinese versions of the Stroop tasks. In the first condition, the colors of the fonts shown on the screen matched with the color name shown (congruent task). In the second condition, the colors of fonts were different from the color name being shown (incongruent task). We recorded the time each subject took to press the key after the presentation of a font on the monitor (RTs) and entered data only when subjects made the correct response. In addition, we recorded the error responses that subjects made as a measure of accuracy. We averaged students’ RTs from the congruent and the incongruent trials separately. We also recorded the number of incorrect responses students’ made. Only trials with correct responses were used in the data analysis.

### DTI Acquisition

Diffusion-tensor imaging data were acquired on a Philips 3T Achieva scanner (v3.26) using an eight-channel head coil. An echo-planar diffusion spin-echo pulse sequence was used with the following parameters: 64 diffusion-gradient directions, *b*-value = 1,500 mm-2, TR = 8.986 ms, TE = 77 ms, acquisition matrix size 136 × 133 × 76, acquisition voxel size: 1.76 mm × 1.8 mm × 1.8 mm, reconstructed voxel size: 1.5 mm × 1.5 mm × 1.8 mm, EPS factor 47, receiver bandwidth 2,160 Hz, sound pressure 18.46 dB, fold-over direction AP, fat shift direction posterior (P) for TOPUP and anterior (A) for TOPDOWN, slice thickness = 1.8, SENSE factor 3 in the anterior–posterior direction, scan duration 12:12.7 mm × 2 for both TOPUP and TOPDOWN.

### DTI Analysis

The FMRIB Software Library (FSL) 5.0.5 Diffusion Toolbox (FDT^2^) was used to process the DTI data. We used a multiple-step procedure recommended by FSL that includes (1) correcting for motion artifact and eddy current with “eddy” and “topup” toolbox, (2) removing skull and non-brain tissue from the image using the Brain Extraction toolbox, and (3) voxel-by-voxel calculation of the diffusion tensors. FA maps were generated using the DTIFit tool.

All DTI data were examined before and after pre-processing to evaluate image quality. Tract-based spatial statistics (TBSS), available in FSL (Smith et al., 2006) was used to perform voxel-wise statistical analysis. TBSS analysis is comprised of the following steps: (1) non-linear alignment of each subject’s FA volume to a 1 × 1 × 1 standard space, (2) selection of a typical image to use as a group template, (3) non-linear transformation of image volumes previously aligned to the group template to the 1 × 1 × 1 Montreal Neurological Institute (MNI152) space, (3) creation of a mean FA skeleton that represents the center of all tracts common to all subjects, and (4) projection of each subject’s aligned FA image onto the mean FA skeleton to generate a study-specific mean FA map (mean FA). We set the threshold at 0.2 for the mean FA map to generate a white-matter tract skeleton that represented the center of the tracts common to all subjects. We then projected each subject’s FA data onto the FA skeleton (all_FA_skeletonise) for voxel-wise statistical comparison. The “tbss_non_FA” script was then used to obtain an MO map for each study participant. We used the general linear model in the FEAT toolbox with higher-level function. We entered subjects’ RTs in the task as a predictor. All subjects’ RTs were demeaned to have a zero group mean. We used Randomize (Nichols and Holmes, 2002), which utilizes permutation-based non-parametric inferences, to perform statistical analysis on the MO matrix and subjects’ RTs calculated from the congruent, incongruent trials, as well as the difference between the congruent and incongruent trials^3^ (*n* = 5,000). We used the family-wise error rate (FWR) corrected with threshold-free cluster enhancement (TFCE) method^4^ to determine the statistical significance at a *p* level of 0.05.

We used the “Cluster” tool in the FSL to extract brain voxels from the resulting statistical images, and obtained the cluster size and peak coordinates. We used software developed in-house to identify MO values that exceeded the statistical threshold at 0.05 in individuals’ MO maps. We mapped the clusters to the Johns Hopkins University ICBM-DTI-81 white-matter labels atlas and Johns Hopkins University white-matter tractography atlas provided by FSL to identify their anatomical location. We calculated the mean of MO values of each subject by summing the MO values within the cluster and divided the number of voxels within the cluster.

### Statistics

We used the Statistical Toolbox in the MATLAB for statistical analysis. We used a linear regression model where FA was the predictor and students’ RTs in the task were the dependent variable. We used one-sample two-tailed *t*-test analysis to assess whether students’ RTs in the incongruent trials were significantly longer than the ones in the congruent trials. We used the Pearson’s correlation analysis to assess whether students’ RTs in the tasks using English versus Chinese fonts correlated with each other. We applied Bonferroni correction to correct for multiple comparisons.

## Results

### Bilingual Adults Showed Comparable Reaction Times (RTs) to Fonts Printed in Blue, Green, Red, or Magenta

We first examined whether students’ RTs to various font colors significantly differed from one another in the task. Using the analysis of variance analysis (ANOVA), we found that there was no statistical difference in subjects’ RTs in responses to different font colors during congruent condition [*F*_(3,80)_ = 0.5858, *p* = 0.6263, Supplementary Figure S1A]. Similarly, we did not find students’ RTs recorded during the incongruent condition differ by the font colors [*F*_(3,80)_ = 0.8, *p* = 0.4958, Supplementary Figure S1B). To ensure that students’ had robust responses in the congruent and incongruent conditions, we evaluated their correct responses in each condition. We showed that the median of correct response was 50 for congruent condition, and 51 for incongruent condition (Supplementary Figure S2). In order to make sure that students’ responses were reliable in the task, we also examined the rate of accuracy, indexed by the number of correct response over the total number of trial. We found that there was no statistical difference in the rate of accuracy between congruent and incongruent conditions [*t*_(20)_ = 0.5308, *p* = 0.6014]. The median of rate of accuracy was 100% for congruent condition and 98.04% for incongruent condition (Supplementary Figure S3). These results indicate that students’ responses in the tasks were highly reliable. Finally, the total amount of time students’ took to make 100 correct responses in the task ranged between 70 and 130 s, with the maximal number of 105 trials observed in 9.5% of total students (Supplementary Figure S4).

### Higher MO Values in the Right Forceps Minor and Anterior Thalamic Radiation Correlated with Shorter RTs in the Stroop Task Using English Fonts

We used an unbiased whole-brain analysis, TBSS, to investigate whether there was any brain cluster in which MO or FA was correlated with students’ RTs in the Stroop color-naming task. We first investigated words in English (**Figure 1A**). We found one brain cluster in the right forceps minor and anterior thalamic radiation where the MO values were significantly negatively correlated with students’ RTs in the incongruent trials (Family-wise rate with TFCE correction for multiple comparison methods, *p* = 0.05, **Figure 1B**). We found that students’ who showed shorter RTs had larger MO values in the cluster compared to the ones who showed longer RTs (**Figure 1C**). We did not find FA correlated with students’ RTs using the same unbiased whole-brain analysis. Anterior thalamic projection is a projecting fiber pathway connecting the thalamus and the cingulate cortex to the frontal region. On the other hand, forceps minor is a commissure pathway connecting the bilateral frontal regions. Higher MO values suggest a predominant fiber orientation in the observed cluster, which contains both anterior thalamic radiation and forceps minor. Because increased MO and increased FA were shown in a region of crossing fiber, we wanted to know whether students with high MO would have higher FA within the cluster. We used the correlation analysis and found that that MO values were significantly positively correlated with FA (Pearson’s *r* = 0.7177, *p* = 0.0003). This result indicates that students with higher FA also had higher MO, which correlated with shorter RTs, compared to students who had lower FA and MO. This finding supports our hypothesis that a brain fiber pathway connecting the anterior cingulate to the frontal region, together with a brain fiber pathway connecting bilateral frontal regions, is related to performance in the Stroop color-naming task.

Fractional anisotropy has been shown to be important for cognitive functions. In order to understand whether students’ RTs were explained by FA, we performed linear regression analysis using students’ FA values in the cluster as a predictor. The results from the linear regression analysis showed that FA values significantly predicted students’ RTs (*R*^2^ = 0.4529, *p* = 0.0008), and 45.29% of total variance of students’ RTs in the task using the English fonts can be explained by the FA values in this brain region alone (**Figure 1D**). This result is in line with other studies showing that higher FA values predict higher learned skills (Johansen-Berg et al., 2007; Mandl et al., 2008; Schlegel et al., 2012; Qi et al., 2015).

In order to confirm that the observed MO-RT relationship did not differ by the font color, we correlated students’ RT to individual font color with their MO values. The results showed that significant relationships between MO and subjects’ RTs held for all font colors that were used in the task (for MAGENTA: *r* = -0.7437, *p* = 0.0004; for BLUE = -0.6004, *p* = 0.016; for RED = -0.7772, *p* = 0.0001 for GREEN: *r* = -0.6763, *p* = 0.0032; all *p*-values were corrected for multiple comparisons; Supplementary Figure S5).

We used a separate TBSS analysis to investigate whether there were any brain regions where the MO or FA was correlated with subjects’ RTs recorded during the congruent trials. We did not find any DTI index correlated with subjects’ RTs recorded during the congruent trials in the task.

In order to confirm whether the RTs for the incongruent trials were longer than those for the congruent trials as previously shown (MacLeod and MacDonald, 2000), we performed a two-tailed one-sample *t*-test. Indeed, we found that students’ RTs recorded in the incongruent trials (mean = 1.0182, standard deviation = 0.1949) were significantly longer than those recorded in the congruent trials (mean = 0.8295, standard deviation = 0.1473) [*t*_(20)_ = 3.373, *p* = 5.475 × 10^-7^].

### Higher MO Values in the Right Forceps Minor and Anterior Thalamic Radiation Correlated with Students’ RTs in the Stroop Task Using Chinese Characters

In the second task, we presented the same words that were used in the first task, but wrote them in Chinese characters instead (**Figure 2A**). We used a separate TBSS analysis to investigate whether there were any brain clusters where the values of MO or FA were correlated with students’ RTs in the Chinese version of the task. We first examined the RTs recorded in the incongruent trials. We found that the MO values in a brain cluster located in the right forceps minor and anterior thalamic radiation were significantly negatively correlated with students’ RTs in the incongruent trials (Family-wise rate with TFCE correction for multiple comparison method, *p* = 0.05, **Figure 2B**). Students who had shorter RTs also had higher MO values (**Figure 2C**). To examine whether students’ RTs can be predicted by FA, we performed linear regression analysis using FA as a predictor. We found that FA values significantly predicted students’ RTs (*R*^2^ = 0.2078, *p* = 0.0378), and explained 20.78% of total variance in students’ RTs in the Chinese version of the task (**Figure 2D**). This finding is consistent with the result shown in **Figure 1D**. The brain cluster identified using Chinese characters in the Stroop task overlapped substantially with the cluster identified using the English version of the Stroop task.

**FIGURE 3:**
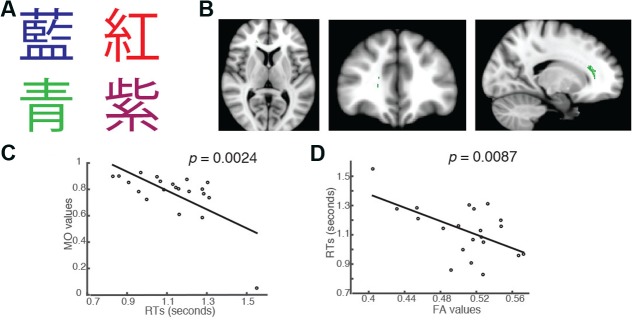
Students’ RTs were significantly negatively correlated with the MO values **(A)** when the color of the fonts matched the named color in congruent trials of the Chinese version of the Stroop task. **(B)** Axial, coronal and horizontal views of a brain cluster (shown in green) where the MO values were significantly negatively correlated with students’ RTs. MNI Coordinates of the peak voxel were: *X*: 18, *Y*: 34, *Z*: 10. The total number of voxels within the cluster was 180. **(C)** A negative relationship between students’ RTs in the congruent trials and the MO values in the brain cluster shown in **(B)**. **(D)** FA values in this brain cluster shown in **(B)** were used in a linear regression model to predict students’ RTs in the congruent condition. Results from the linear regression analysis showed that FA significantly predicted students’ RTs in the congruent conditions and explained 31.07% of the total variance in students’ responses in the task (*R*^2^ = 0.3107, *p* = 0.0087).

In addition to the significant brain-behavior relationship found in the incongruent condition, we also identified a brain cluster where the MO values were significantly negatively correlated with students’ RTs in the Chinese version of the congruent trials (Family-wise rate with TFCE correction for multiple comparison methods, *p* = 0.05; **Figures 3A,B**). This brain cluster also overlapped substantially with the ones shown in **Figures 1B**, **2B**. Subjects who had higher MO values had shorter RTs in the congruent trials (**Figure 3C**). We also performed a linear regression analysis using FA as a predictor. The analysis showed that FA values significantly predicted students’ RTs in the trials and explained 31.07% of the total variance (*R*^2^ = 0.3107, *p* = 0.0087, **Figure 3D**).

We also examined whether the RTs in the incongruent trials differed significantly from those in the congruent trials as we observed in the English version of the Stroop task. We found that the RTs in the incongruent trials (mean = 1.2299, standard deviation = 0.1781) were significantly longer than those in the congruent trials (mean = 1.1299, standard deviation = 0.1760) [two-tailed paired *t*-test, *t*_(20)_ = 3.3725, *p* = 0.003], consistent with our finding in English version of the task and with results reported by other researchers (MacLeod and MacDonald, 2000).

Finally, we examined whether there was a difference in subjects’ RTs observed between the English and Chinese versions of the Stroop task. We used the two-tailed one-sample *t*-test and compared the mean of RTs in both tasks. We found that the mean of subjects’ RTs in the Chinese version of the Stroop task was significantly longer than the mean of RTs recorded in the English version of the Stroop task [*t*_(20)_ = -8.8171, *p* = 2.51 × 10^-8^, Supplementary Figure S6]. This result is in line with the literature that bilingual individuals required longer time to respond in a task where fonts were printed in their first language following a task where fonts were printed in their second language (Costa and Santesteban, 2004; Branzi et al., 2016).

### RTs in the Task Using English Words Correlated with RTs in the Same Task Using Chinese Characters

Because we observed a great degree of overlap in the brain clusters identified using English and Chinese versions of the task, we expected that students’ RTs recorded in these two tasks would correlate with one another. Indeed, we found that students’ RTs for incongruent trials of the English and Chinese versions of the task were significantly positively correlated (Pearson’s *r* = 0.8296, *p* = 6.17 × 10^-6^, corrected for multiple comparisons). Similarly, students’ RTs for the congruent trials of the English and Chinese versions of the task were significantly correlated (Pearson’s *r* = 0.4959, *p* = 0.04, corrected for multiple comparisons). Students who showed longer RTs in the English Stroop also showed longer RTs in the Chinese Stroop (English Stroop: mean_incongruent_ = 1.02 s, standard deviation_incongruent_ = 0.195; mean_congruent_ = 0.83 s, standard deviation_congruent_ = 0.147; Chinese Stroop: mean_incongruent_ = 1.23, standard deviation_incongruent_ = 0.178; mean_congruent_ = 1.13, standard deviation = 0.176).

Taken together, these observations suggest that students’ RTs in the Stroop color-naming task may depend on the organization of two brain fiber pathways, forceps minor and anterior thalamic radiation, in the right frontal region. Using the MO, a DTI index of the shape of diffusion tensor depending on crossing fiber, we showed that young bilingual adults with higher MO values in the right frontal region showed shorter RTs compared to individuals with lower MO values. This result supports our hypothesis that a brain fiber pathway connecting the ACC and striatum to the anterior frontal region, together with a brain fiber pathway connecting the bilateral frontal regions, were related to bilinguals’ performances in the EF task. Furthermore, the anterior thalamic radiation and forceps minor conjunction at the right frontal region highlights the importance of fiber crossing in relation to EF skill. Importantly, we provided a novel observation that the relationship between the MO and students’ RTs was found not only in the task using English words but also in the task using Chinese characters, suggesting that this brain signature is important for the control of attention in bilinguals using either their first or their second language.

## Discussion

In the present study, we investigated whether the structural properties of brain fiber pathways that connect ACC and striatum to the anterior frontal region can predict bilingual college students’ RTs in the color-naming version of the Stroop task. We found that in Chinese–English bilingual college students, the structural properties of right forceps minor and anterior thalamic radiation predicted their RTs in tasks that measured their attention-control skills. Students with higher MO and FA values showed shorter RTs when the fonts displayed did not match the names of colors compared to individuals with lower MO and FA values in the same region. Importantly, the same relationship was found regardless of the language, English or Chinese, in which the words were printed. Students who were better at responding to fonts printed in their first language were also better at responding to fonts printed in their second language. Taken together, our findings suggest that the right forceps minor and anterior thalamic radiation may serve as an attention control center that enables bilinguals to direct their attention to a targeted presentation shown in their first or second language.

### Highly Organized Right Forceps Minor and Anterior Thalamic Radiation Can Predict Higher Attention Control Skills

The observed relationship between the MO values in the right forceps minor and anterior thalamic radiation and students’ RTs suggest that students were faster and better at responding to the printed fonts when there was a predominant fiber pathway in the right frontal region. As previously described, a voxel would have MO and FA values close to 1 when all fibers in that voxel are oriented in a predominant direction. FA, a common DTI index, has been associated with various cognitive functions. For example, college students who show higher FA values in fiber pathways connecting frontal and posterior language areas had better second language learning outcomes than the ones who had lower FA in the same region (Qi et al., 2015; Mamiya et al., 2016). Furthermore, bilingual adults and elderly subjects show higher FA values in the corpus callosum, SLF, and IFOF compared to their monolingual counterparts, suggesting bilingualism may increase FA values in these regions (Luk et al., 2011; Pliatsikas et al., 2015). The relationship we found between MO/FA and students’ RTs suggests that the organization of right forceps minor and anterior thalamic radiation can affect how fast individuals respond in tasks that require attention control.

The forceps minor, the anterior part of the corpus callosum, connects the homologous regions of the anterior frontal lobes between two hemispheres [for reviews, see (Paul et al., 2007; Fame et al., 2011; Fabri et al., 2014)]. Among the regions connected, the frontopolar cortex has been shown to be important for cognitive behaviors in human and non-human primates (Semendeferi et al., 2001; Boschin et al., 2015). It has been shown that neural activity recorded in this region corresponds to animals’ decision making during feedback learning (Tsujimoto et al., 2010). In human patients, the forceps minor is implicated in cognitive dysfunctions. For example, it has been shown that patients with frontotemporal dementia have lower FA values in the forceps minor than healthy subjects (Lillo et al., 2012). It has also been shown that FA values in the forceps minor can be used in a binary classification algorithm in machine learning to predict cognitive impairments in patients (Haller et al., 2010). Similarly, children with attention-deficit-hyperactivity disorder (ADHD) show lower FA values in the forceps minor than healthy individuals (Qiu et al., 2011). In healthy human subjects, the right frontopolar cortex showed brain activity when subjects named the font colors in the incongruent trials in the Stroop task (Bench et al., 1993; Carter et al., 1995). Findings from these studies using human and animals suggest that neural activity in the anterior frontal region is involved in the control of attention. Subjects who showed higher diffusion properties of brain fiber pathway connecting bilateral anterior frontal regions had better control of attention skills than individuals with lower diffusion properties.

In addition to the forceps minor, MO in the anterior thalamic radiation was correlated with students’ RTs in the tasks. Anterior thalamic radiation connects the anterior and ventromedial nuclei of thalamus to the prefrontal cortex, including anterior cingulate and dorsolateral frontal regions (Shibata, 1993; Shibata and Naito, 2005; Wright et al., 2013). These brain regions are central to the attention-control network [for reviews, see (MacLeod and MacDonald, 2000; Menon and Uddin, 2010; Leszczynski and Staudigl, 2016)]. The anterior cingulate and dorsolateral prefrontal cortex have long been associated with human cognition. For examples, the anterior cingulate shows activity when subjects name the font colors in the Stroop task (Pardo et al., 1990; Bench et al., 1993; Carter et al., 1995; Leung et al., 2000). Recently, the role of anterior thalamus in attention-control has been demonstrated in rodents and humans. Wimmer et al. (2015) showed that the feedforward information in the thalamocortical connection affects whether sensory or auditory input is selected and used in the task (Wimmer et al., 2015). Animals with lesions in the anterior thalamus failed to attend to the predictors specifically associated with rewards (Wright et al., 2015), suggesting that the anterior thalamus may be associated with working memory. Consistent with this idea, de Bourbon-Teles et al. (2014) used functional MR imaging and revealed that healthy human adults showed activity in the anterior thalamus when they use predictors that have been previously learned to guide their attention in a working memory task (de Bourbon-Teles et al., 2014). In the present study, we observed that higher MO and FA values in the anterior thalamic radiation predict better attention control skills. This finding suggests that in subjects with better attention control skills, fiber pathways connecting the anterior and ventromedial nuclei of thalamus to the prefrontal cortex have tissue properties that enable better information flow across brain regions.

The students’ FA values observed in the study are consistent with the FA values reported in the literature (Yeatman et al., 2012; Fabri et al., 2014). Among children and adolescents, FA values ranged from 0.4 to 0.6 in the forceps minor and the anterior portion of the corpus callosum. It is thought that the variability of FA values may be related to fiber crossing. In the present study, we identified a brain cluster that contained forceps minor and anterior thalamic radiation. The forceps minor is a part of the largest commissural fiber pathway connecting bilateral anterior frontal regions (Wakana et al., 2004; Catani and Thiebaut de Schotten, 2008). On the other hand, anterior thalamic radiation ascends from the brainstem and connects thalamus, striatum and ACC to the anterior frontal region. Higher FA and MO values in the right frontal region suggest that a predominant fiber orientation in that region is better for bilinguals’ responses to fonts printed in their first or second languages. It is possible that the neuroanatomical properties of these two brain fiber pathways are related to students’ second language learning experiences because previous studies have shown that learning can induce structural plasticity in developing and adult human brains [for reviews, see (Fields, 2008; Zatorre et al., 2012)]. When bilingual individuals choose which language to use, and inhibit the use of the second language, anterior frontal regions exert executive control over brain activities in the ACC, caudate nucleus, putamen, and thalamus. The implication is that the forceps minor may be the predominant fiber direction allowing better executive control. Anterior thalamic radiation may be the secondary fiber pathway crossing the forceps minor. However, the current analysis cannot determine whether or not the forceps minor is the predominant fiber pathway. Further studies are required to determine how forceps minor and anterior thalamic radiation crossing gives rise to various EF skills in bilingual individuals.

Taken together, the observed relationship between the MO values in the right forceps minor/anterior thalamic radiation and students’ RTs in the tasks suggests that information from the thalamus and anterior cingulate, as well as from the anterior frontal region, jointly affect students’ abilities to attend to words printed in their first and second languages. When bilinguals switch between two languages, the structural connectivity between the brain cluster observed and the posterior language areas may be strengthened. Consistent with this hypothesis, two recent reports by Luk et al. (2011) and Pliatsikas et al. (2015) have shown that bilingual adults and elderly people have higher FA values in the corpus callosum and superior longitudinal fasciculus compared to control subjects (Luk et al., 2011; Pliatsikas et al., 2015).

There are some limitations to this study. First, lower MO values assume that two fiber bundles in a given voxel are organized and oriented differently. This does not take into account the possibility of having three or more fiber bundles in a voxel. Furthermore, MO values may decrease when a fiber bundle fans out. In the case of the corpus callosum, fiber pathways are well-bundled and highly organized in a medial-lateral direction along the mid-sagittal plane. As fiber pathways enter the designated cortical sites, they begin to fan out, which can lower MO. Despite this limitation, we found that the MO values range between 0.75 and 0.98, and the FA values in the same cluster range between 0.4 and 0.6, suggesting that a single dominant fiber bundle is likely to be present in these voxels. In the future, it will be interesting to use different diffusion-weighted modeling techniques to study whether there is a relationship between students’ RTs in the color-naming Stroop task and fiber crossing in the right forceps minor and anterior thalamic radiation.

The second limitation of the study is that it does not consider genetic influences on executive-function skills. Friedman et al. (2008) used twins to show that there are strong genetic influences on how well subjects perform in executive function tasks. Another study by Sakai et al. (2004) also used twins to show that brain activity observed during second language learning is under the influence of genetic factors. More recent studies using gene-behavior association analysis have also identified genetic factors that affect human cognitive functions. However, the results from association studies are not always consistent [for examples, (Bruder et al., 2005; Barnett et al., 2008; Goldman et al., 2009; Wardle et al., 2013)]. One plausible explanation may be the presence of an interaction effect between different genetic factors, or between genetic factors and brain structures, which can lead to different behavioral results. Consistent with this idea, we recently demonstrated that polymorphisms in the *COMT* gene can affect the relationship between FA values and students’ learning from exposure to a second language learning environment (Mamiya et al., 2016), suggesting that it may be necessary to take into account genetic factors when looking for structural correlates of cognitive functions.

In summary, we used behavioral and whole-brain analyses to show that (1) students’ MO values in the right forceps minor and anterior thalamic radiation correlate with their RTs in color-naming version of the Stroop task, (2) the same relationship was observed when we presented fonts printed in students’ first or second language, (3) higher FA values in the same brain clusters also predicted shorter RTs in the task, and finally (4) students who had shorter RTs in the English Stroop task also had shorter RTs in the Chinese Stroop task. These results suggest that the right forceps minor and anterior thalamic radiation are involved when bilinguals direct their attention to a specific task, such as when words are presented in two different languages.

## Ethics Statement

This study was carried out in accordance with the recommendations of the University of Washington’s Human Study Section guidelines with written informed consent from all subjects in accordance with the Declaration of Helsinki. The protocol was approved by the Committee D/E.

## Author Contributions

PM designed and conducted the study; PM and TR analyzed the data; PM, TR, and PKK wrote the manuscript.

## Conflict of Interest Statement

The authors declare that the research was conducted in the absence of any commercial or financial relationships that could be construed as a potential conflict of interest.

## Footnotes

1 https://fsl.fmrib.ox.ac.uk/fslcourse/physics+apps/Diffusion_analysis_interpretation.pdf

2 http://www.fmrib.ox.ac.uk/fsl/fdt/

3 http://www.fmrib.ox.ac.uk/fsl/randomise/

4 http://www.fmrib.ox.ac.uk/analysis/research/tfce

## References

[B1] Barnett J. H. Scoriels L. Munafo M. R. (2008). Meta-analysis of the cognitive effects of the catechol-O-methyltransferase gene Val158/108Met polymorphism. *Biol. Psychiatry* 64 137–144. 10.1016/j.biopsych.2008.01.005 18339359

[B2] Bench C. J. Frith C. D. Grasby P. M. Friston K. J. Paulesu E. Frackowiak R. S. (1993). Investigations of the functional anatomy of attention using the Stroop test. *Neuropsychologia* 31 907–922. 10.1016/0028-3932(93)90147-R 8232848

[B3] Bialystok E. Craik F. Luk G. (2008). Cognitive control and lexical access in younger and older bilinguals. *J. Exp. Psychol. Learn. Mem. Cogn.* 34 859–873. 10.1037/0278-7393.34.4.859 18605874

[B4] Bialystok E. Craik F. I. Luk G. (2012). Bilingualism: consequences for mind and brain. *Trends Cogn. Sci.* 16 240–250. 10.1016/j.tics.2012.03.001 22464592 PMC3322418

[B5] Bialystok E. Martin M. M. Viswanathan M. (2005). Bilingualism across the lifespan: the rise and fall of inhibitory control. *Int. J. Biling.* 9 103–119.

[B6] Boschin E. A. Piekema C. Buckley M. J. (2015). Essential functions of primate frontopolar cortex in cognition. *Proc. Natl. Acad. Sci. U.S.A.* 112 E1020–E1027. 10.1073/pnas.1419649112 25691741 PMC4352768

[B7] Branzi F. M. Della Rosa P. A. Canini M. Costa A. Abutalebi J. (2016). Language control in bilinguals: monitoring and response selection. *Cereb. Cortex* 26 2367–2380. 10.1093/cercor/bhv052 25838037

[B8] Bruder G. E. Keilp J. G. Xu H. Shikhman M. Schori E. Gorman J. M. (2005). Catechol-O-methyltransferase (COMT) genotypes and working memory: associations with differing cognitive operations. *Biol. Psychiatry* 58 901–907. 10.1016/j.biopsych.2005.05.010 16043133

[B9] Carter C. S. Mintun M. Cohen J. D. (1995). Interference and facilitation effects during selective attention: an H215O PET study of Stroop task performance. *Neuroimage* 2 264–272. 10.1006/nimg.1995.1034 9343611

[B10] Catani M. Thiebaut de Schotten M. (2008). A diffusion tensor imaging tractography atlas for virtual in vivo dissections. *Cortex* 44 1105–1132. 10.1016/j.cortex.2008.05.004 18619589

[B11] Coderre E. L. van Heuven W. J. (2014). The effect of script similarity on executive control in bilinguals. *Front. Psychol.* 5:1070. 10.3389/fpsyg.2014.01070 25400594 PMC4212224

[B12] Costa A. Hernandez M. Sebastian-Galles N. (2008). Bilingualism aids conflict resolution: evidence from the ANT task. *Cognition* 106 59–86. 10.1016/j.cognition.2006.12.013 17275801

[B13] Costa A. Santesteban M. (2004). Lexical access in bilingual speech production: evidence from language switching in highly proficient bilinguals and L2 learners. *J. Mem. Lang.* 50 491–511. 10.1016/j.jml.2004.02.002

[B14] Costa A. Sebastian-Galles N. (2014). How does the bilingual experience sculpt the brain? *Nat. Rev. Neurosci.* 15 336–345. 10.1038/nrn3709 24739788 PMC4295724

[B15] Cummine J. Boliek C. A. (2013). Understanding white matter integrity stability for bilinguals on language status and reading performance. *Brain Struct. Funct.* 218 595–601. 10.1007/s00429-012-0466-6 23097036

[B16] de Bourbon-Teles J. Bentley P. Koshino S. Shah K. Dutta A. Malhotra P. (2014). Thalamic control of human attention driven by memory and learning. *Curr. Biol.* 24 993–999. 10.1016/j.cub.2014.03.024 24746799 PMC4012133

[B17] Douaud G. Jbabdi S. Behrens T. E. Menke R. A. Gass A. Monsch A. U. (2011). DTI measures in crossing-fibre areas: increased diffusion anisotropy reveals early white matter alteration in MCI and mild Alzheimer’s disease. *Neuroimage* 55 880–890. 10.1016/j.neuroimage.2010.12.008 21182970 PMC7116583

[B18] Fabri M. Pierpaoli C. Barbaresi P. Polonara G. (2014). Functional topography of the corpus callosum investigated by DTI and fMRI. *World J. Radiol.* 6 895–906. 10.4329/wjr.v6.i12.895 25550994 PMC4278150

[B19] Fame R. M. Macdonald J. L. Macklis J. D. (2011). Development, specification, and diversity of callosal projection neurons. *Trends Neurosci.* 34 41–50. 10.1016/j.tins.2010.10.002 21129791 PMC3053014

[B20] Fields R. D. (2008). White matter in learning, cognition and psychiatric disorders. *Trends Neurosci.* 31 361–370. 10.1016/j.tins.2008.04.001 18538868 PMC2486416

[B21] Friedman N. P. Miyake A. Young S. E. Defries J. C. Corley R. P. Hewitt J. K. (2008). Individual differences in executive functions are almost entirely genetic in origin. *J. Exp. Psychol. Gen.* 137 201–225. 10.1037/0096-3445.137.2.201 18473654 PMC2762790

[B22] Gold B. T. Johnson N. F. Powell D. K. (2013). Lifelong bilingualism contributes to cognitive reserve against white matter integrity declines in aging. *Neuropsychologia* 51 2841–2846. 10.1016/j.neuropsychologia.2013.09.037 24103400 PMC3856701

[B23] Goldman D. Weinberger D. R. Malhotra A. K. Goldberg T. E. (2009). The role of COMT Val158Met in cognition. *Biol. Psychiatry* 65 e1–e2; author reply e3–e4. 10.1016/j.biopsych.2008.07.032 18838132 PMC2679368

[B24] Grundy J. G. Anderson J. A. E. Bialystok E. (2017). Neural correlates of cognitive processing in monolinguals and bilinguals. *Ann. N. Y. Acad. Sci.* 1396 183–201. 10.1111/nyas.13333 28415142 PMC5446278

[B25] Haller S. Nguyen D. Rodriguez C. Emch J. Gold G. Bartsch A. (2010). Individual prediction of cognitive decline in mild cognitive impairment using support vector machine-based analysis of diffusion tensor imaging data. *J. Alzheimers Dis.* 22 315–327. 10.3233/JAD-2010-100840 20847435

[B26] Jbabdi S. Behrens T. E. Smith S. M. (2010). Crossing fibres in tract-based spatial statistics. *Neuroimage* 49 249–256. 10.1016/j.neuroimage.2009.08.039 19712743

[B27] Johansen-Berg H. Della-Maggiore V. Behrens T. E. Smith S. M. Paus T. (2007). Integrity of white matter in the corpus callosum correlates with bimanual co-ordination skills. *Neuroimage* 36(Suppl. 2), T16–T21. 10.1016/j.neuroimage.2007.03.041 17499163 PMC3119816

[B28] Kuhl P. K. Stevenson J. Corrigan N. M. Van Den Bosch J. J. F. Can D. D. Richards T. (2016). Neuroimaging of the bilingual brain: structural brain correlates of listening and speaking in a second language. *Brain Lang.* 162 1–9. 10.1016/j.bandl.2016.07.004 27490686

[B29] Leszczynski M. Staudigl T. (2016). Memory-guided attention in the anterior thalamus. *Neurosci. Biobehav. Rev.* 66 163–165. 10.1016/j.neubiorev.2016.04.015 27130694

[B30] Leung H. C. Skudlarski P. Gatenby J. C. Peterson B. S. Gore J. C. (2000). An event-related functional MRI study of the stroop color word interference task. *Cereb. Cortex* 10 552–560. 10.1093/cercor/10.6.552 10859133

[B31] Lillo P. Mioshi E. Burrell J. R. Kiernan M. C. Hodges J. R. Hornberger M. (2012). Grey and white matter changes across the amyotrophic lateral sclerosis-frontotemporal dementia continuum. *PLOS ONE* 7:e43993. 10.1371/journal.pone.0043993 22952843 PMC3430626

[B32] Luk G. Bialystok E. Craik F. I. Grady C. L. (2011). Lifelong bilingualism maintains white matter integrity in older adults. *J. Neurosci.* 31 16808–16813. 10.1523/JNEUROSCI.4563-11.2011 22090506 PMC3259110

[B33] MacLeod C. M. MacDonald P. A. (2000). Interdimensional interference in the Stroop effect: uncovering the cognitive and neural anatomy of attention. *Trends Cogn. Sci.* 4 383–391. 10.1016/S1364-6613(00)01530-8 11025281

[B34] Mamiya P. C. Richards T. L. Coe B. P. Eichler E. E. Kuhl P. K. (2016). Brain white matter structure and COMT gene are linked to second-language learning in adults. *Proc. Natl. Acad. Sci. U.S.A.* 113 7249–7254. 10.1073/pnas.1606602113 27298360 PMC4932981

[B35] Mandl R. C. Schnack H. G. Zwiers M. P. van der Schaaf A. Kahn R. S. Hulshoff Pol H. E. (2008). Functional diffusion tensor imaging: measuring task-related fractional anisotropy changes in the human brain along white matter tracts. *PLOS ONE* 3:e3631. 10.1371/journal.pone.0003631 18982065 PMC2574009

[B36] Martin-Rhee M. M. Bialystok E. (2008). The development of two types of inhibitory control in monolingual and bilingual children. *Bilingualism* 11 81–93. 10.1017/S1366728907003227 25935936

[B37] Menon V. Uddin L. Q. (2010). Saliency, switching, attention and control: a network model of insula function. *Brain Struct. Funct.* 214 655–667. 10.1007/s00429-010-0262-0 20512370 PMC2899886

[B38] Mohades S. G. Struys E. Van Schuerbeek P. Mondt K. Van De Craen P. Luypaert R. (2012). DTI reveals structural differences in white matter tracts between bilingual and monolingual children. *Brain Res.* 1435 72–80. 10.1016/j.brainres.2011.12.005 22197702

[B39] Mohades S. G. Van Schuerbeek P. Rosseel Y. Van De Craen P. Luypaert R. Baeken C. (2015). White-matter development is different in bilingual and monolingual children: a longitudinal DTI study. *PLOS ONE* 10:e0117968. 10.1371/journal.pone.0117968 25706865 PMC4338107

[B40] Nichols T. E. Holmes A. P. (2002). Nonparametric permutation tests for functional neuroimaging: a primer with examples. *Hum. Brain Mapp.* 15 1–25. 10.1002/hbm.1058 11747097 PMC6871862

[B41] Pardo J. V. Pardo P. J. Janer K. W. Raichle M. E. (1990). The anterior cingulate cortex mediates processing selection in the Stroop attentional conflict paradigm. *Proc. Natl. Acad. Sci. U.S.A.* 87 256–259. 10.1073/pnas.87.1.256 2296583 PMC53241

[B42] Paul L. K. Brown W. S. Adolphs R. Tyszka J. M. Richards L. J. Mukherjee P. (2007). Agenesis of the corpus callosum: genetic, developmental and functional aspects of connectivity. *Nat. Rev. Neurosci.* 8 287–299. 10.1038/nrn2107 17375041

[B43] Pliatsikas C. Moschopoulou E. Saddy J. D. (2015). The effects of bilingualism on the white matter structure of the brain. *Proc. Natl. Acad. Sci. U.S.A.* 112 1334–1337. 10.1073/pnas.1414183112 25583505 PMC4321232

[B44] Qi Z. Han M. Garel K. San Chen E. Gabrieli J. D. E. (2015). White-matter structure in the right hemisphere predicts Mandarin Chinese learning success. *J. Neurolinguist.* 33 14–28. 10.1016/j.jneuroling.2014.08.004

[B45] Qiu M. G. Ye Z. Li Q. Y. Liu G. J. Xie B. Wang J. (2011). Changes of brain structure and function in ADHD children. *Brain Topogr.* 24 243–252. 10.1007/s10548-010-0168-4 21191807

[B46] Radua J. Grau M. Van Den Heuvel O. A. Thiebaut de Schotten M. Stein D. J. Canales-Rodriguez E. J. (2014). Multimodal voxel-based meta-analysis of white matter abnormalities in obsessive-compulsive disorder. *Neuropsychopharmacology* 39 1547–1557. 10.1038/npp.2014.5 24407265 PMC4023155

[B47] Sakai K. L. Miura K. Narafu N. Muraishi Y. (2004). Correlated functional changes of the prefrontal cortex in twins induced by classroom education of second language. *Cereb. Cortex* 14 1233–1239. 10.1093/cercor/bhh084 15142962

[B48] Schlegel A. A. Rudelson J. J. Tse P. U. (2012). White matter structure changes as adults learn a second language. *J. Cogn. Neurosci.* 24 1664–1670. 10.1162/jocn_a_00240 22571459

[B49] Semendeferi K. Armstrong E. Schleicher A. Zilles K. Van Hoesen G. W. (2001). Prefrontal cortex in humans and apes: a comparative study of area 10. *Am. J. Phys. Anthropol.* 114 224–241. 10.1002/1096-8644(200103)114:3<224::AID-AJPA1022>3.0.CO;2-I 11241188

[B50] Shibata H. (1993). Efferent projections from the anterior thalamic nuclei to the cingulate cortex in the rat. *J. Comp. Neurol.* 330 533–542. 10.1002/cne.903300409 8320343

[B51] Shibata H. Naito J. (2005). Organization of anterior cingulate and frontal cortical projections to the anterior and laterodorsal thalamic nuclei in the rat. *Brain Res.* 1059 93–103. 10.1016/j.brainres.2005.08.025 16157311

[B52] Smith S. M. Jenkinson M. Johansen-Berg H. Rueckert D. Nichols T. E. Mackay C. E. (2006). Tract-based spatial statistics: voxelwise analysis of multi-subject diffusion data. *Neuroimage* 31 1487–1505. 10.1016/j.neuroimage.2006.02.024 16624579

[B53] Stroop J. R. (1935). Studies of interference in serial verbal reactions. *J. Exp. Psychol.* 18 643–662. 10.1037/h0054651

[B54] Takeuchi H. Taki Y. Sassa Y. Hashizume H. Sekiguchi A. Nagase T. (2012). Regional gray and white matter volume associated with Stroop interference: evidence from voxel-based morphometry. *Neuroimage* 59 2899–2907. 10.1016/j.neuroimage.2011.09.064 21988892

[B55] Tamnes C. K. Fjell A. M. Westlye L. T. Ostby Y. Walhovd K. B. (2012). Becoming consistent: developmental reductions in intraindividual variability in reaction time are related to white matter integrity. *J. Neurosci.* 32 972–982. 10.1523/JNEUROSCI.4779-11.2012 22262895 PMC6621149

[B56] Tsujimoto S. Genovesio A. Wise S. P. (2010). Evaluating self-generated decisions in frontal pole cortex of monkeys. *Nat. Neurosci.* 13 120–126. 10.1038/nn.2453 19966838 PMC2834888

[B57] Wakana S. Jiang H. Nagae-Poetscher L. M. Van Zijl P. C. Mori S. (2004). Fiber tract-based atlas of human white matter anatomy. *Radiology* 230 77–87. 10.1148/radiol.2301021640 14645885

[B58] Wardle M. C. De Wit H. Penton-Voak I. Lewis G. Munafo M. R. (2013). Lack of association between COMT and working memory in a population-based cohort of healthy young adults. *Neuropsychopharmacology* 38 k1253–1263. 10.1038/npp.2013.24 23337869 PMC3656369

[B59] Wimmer R. D. Schmitt L. I. Davidson T. J. Nakajima M. Deisseroth K. Halassa M. M. (2015). Thalamic control of sensory selection in divided attention. *Nature* 526 705–709. 10.1038/nature15398 26503050 PMC4626291

[B60] Wright N. F. Vann S. D. Aggleton J. P. Nelson A. J. (2015). A critical role for the anterior thalamus in directing attention to task-relevant stimuli. *J. Neurosci.* 35 5480–5488. 10.1523/JNEUROSCI.4945-14.2015 25855166 PMC4388916

[B61] Wright N. F. Vann S. D. Erichsen J. T. O’mara S. M. Aggleton J. P. (2013). Segregation of parallel inputs to the anteromedial and anteroventral thalamic nuclei of the rat. *J. Comp. Neurol.* 521 2966–2986. 10.1002/cne.23325 23504917 PMC4299679

[B62] Yang S. Yang H. Lust B. (2011). Early childhood bilingualism leads to advances in executive attention: dissociating culture and language. *Bilingualism* 14 412–422. 10.1017/S1366728910000611

[B63] Yeatman J. D. Dougherty R. F. Myall N. J. Wandell B. A. Feldman H. M. (2012). Tract profiles of white matter properties: automating fiber-tract quantification. *PLOS ONE* 7:e49790. 10.1371/journal.pone.0049790 23166771 PMC3498174

[B64] Zatorre R. J. Fields R. D. Johansen-Berg H. (2012). Plasticity in gray and white: neuroimaging changes in brain structure during learning. *Nat. Neurosci.* 15 528–536. 10.1038/nn.3045 22426254 PMC3660656

