# Supplementary material for: Right Forceps Minor and Anterior Thalamic Radiation Predict Executive Function Skills in Young Bilingual Adults
Source: Front Psychol. 2018 Feb 9;9:118. doi: 10.3389/fpsyg.2018.00118 (PMC5811666; doi:10.3389/fpsyg.2018.00118)
Supplement: Supplementary file 1 [file Image_1.pdf]

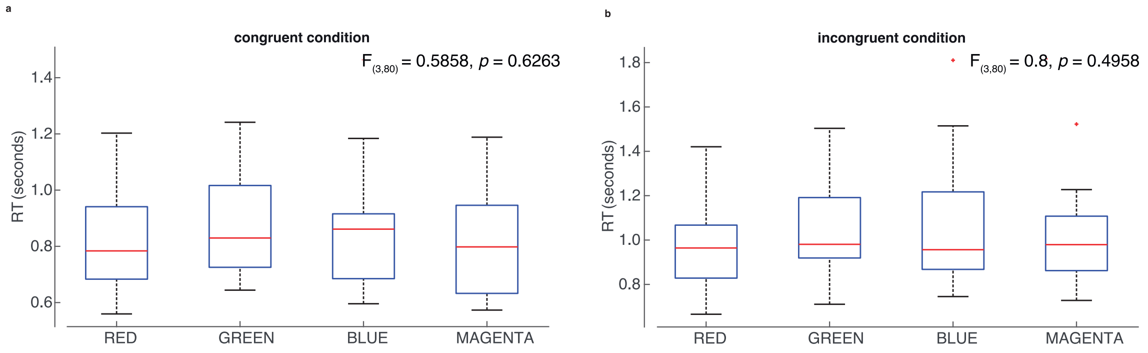

Figure S1. Students' RTs for different colors of fonts recorded during congruent and incongruent conditions. The results from the ANOVA showed no statistical difference in students' RTs in response to different font colors during a) congruent condition ($F_{(3,80)}$ = 0.5858, p = 0.6263), as well as b) incongruent condition ($F_{(3,80)}$ = 0.8, p = 0.4958). Red bars represent the median of students' RTs towards individual font colors. Upper blue line represents the 75th percentile of students' RTs, and the lower blue lines represent the 25th percentile of subjects' RTs. Upper whiskers represent the greatest RTs observed and the lower whiskers represent the smallest RTs observed.
