# Supplementary material for: Right Forceps Minor and Anterior Thalamic Radiation Predict Executive Function Skills in Young Bilingual Adults
Source: Front Psychol. 2018 Feb 9;9:118. doi: 10.3389/fpsyg.2018.00118 (PMC5811666; doi:10.3389/fpsyg.2018.00118)
Supplement: Supplementary file 2 [file Image_2.pdf]

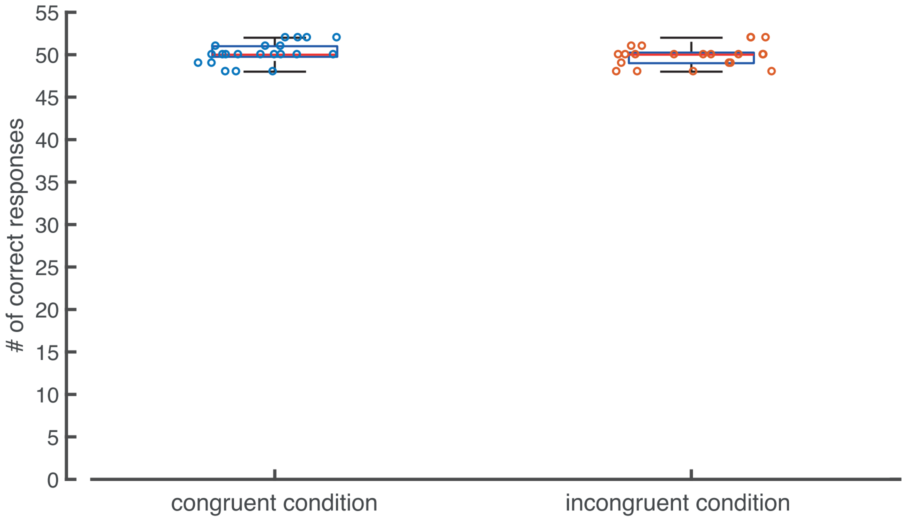


**Figure S2**. The number of correct responses students' made in congruent versus incongruent conditions. Red horizontal bars of the boxplots represent the median. The upper line of the box represents the 75th percentile and the lower line of the box represents the 25th percentile of the number of correct responses. Upper whiskers represent the greatest observation and the lower whiskers represent the smaller observation in each task. Open circles represent the individual data points that are shown in blue for congruent and red for incongruent conditions.
