# Supplementary material for: Right Forceps Minor and Anterior Thalamic Radiation Predict Executive Function Skills in Young Bilingual Adults
Source: Front Psychol. 2018 Feb 9;9:118. doi: 10.3389/fpsyg.2018.00118 (PMC5811666; doi:10.3389/fpsyg.2018.00118)
Supplement: Supplementary file 3 [file Image_3.pdf]

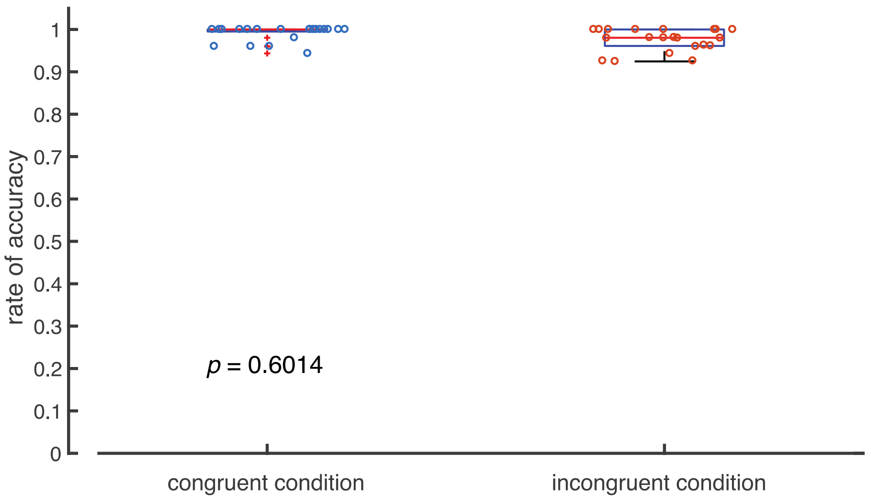

Figure S3. Rates of accuracy in congruent and incongruent conditions. Red lines of the boxplots represent the median rates of accuracy and the lower whiskers represent the smallest rates of accuracy observed in the tasks. Open circles represent the individual data points that are shown in blue for congruent and red for incongruent condition. Y-axis represents the number of correct responses over the total number of trials in each task, indexed as the rate of accuracy. X-axis represents two different conditions, congruent versus incongruent. The results from the one sample paired t-test showed no statistical difference in the rate of accuracy observed between congruent and incongruent conditions ($t_{(20)} = 0.5308$, $p = 0.6014$).
