# Supplementary material for: Right Forceps Minor and Anterior Thalamic Radiation Predict Executive Function Skills in Young Bilingual Adults
Source: Front Psychol. 2018 Feb 9;9:118. doi: 10.3389/fpsyg.2018.00118 (PMC5811666; doi:10.3389/fpsyg.2018.00118)
Supplement: Supplementary file 4 [file Image_4.pdf]

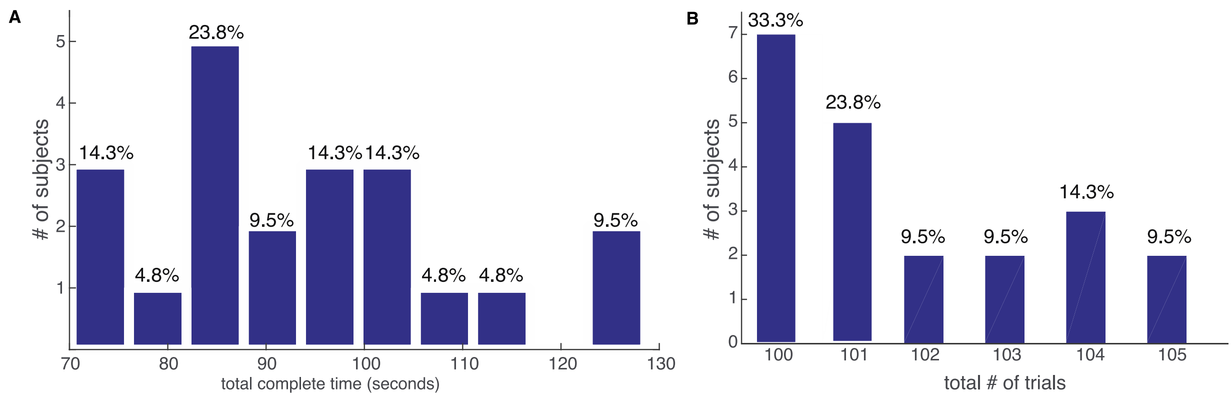

Figure S4. Distributions of students' (A) total complete time, and (B) total number of trials to completion of 100 correct trials. Bars represent the number of subjects. Percentages represent the number of subjects observed in a given bar over the total number of students (n=21).
