# Supplementary material for: Right Forceps Minor and Anterior Thalamic Radiation Predict Executive Function Skills in Young Bilingual Adults
Source: Front Psychol. 2018 Feb 9;9:118. doi: 10.3389/fpsyg.2018.00118 (PMC5811666; doi:10.3389/fpsyg.2018.00118)
Supplement: Supplementary file 5 [file Image_5.pdf]

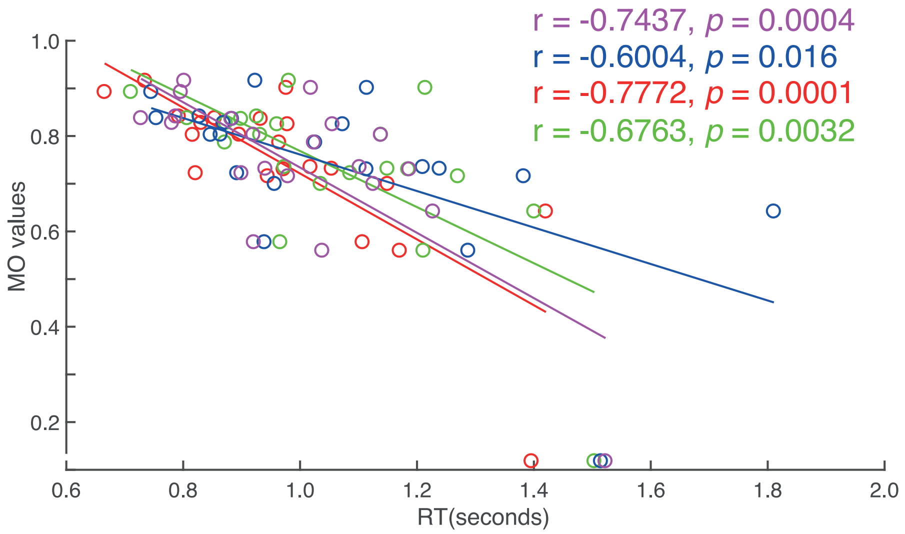

Figure S5. Relationships between the MO values and students' RTs to different font colors. The results from Pearson's correlation analysis revealed that there was no difference in the relationship between the MO values and students' RTs to different font colors. Different colors of open circles represent four different font colors (magenta, blue, red and green) used in the task.
