# Supplementary figures and images for: Right Forceps Minor and Anterior Thalamic Radiation Predict Executive Function Skills in Young Bilingual Adults
Source: Front Psychol. 2018 Feb 9;9:118. doi: 10.3389/fpsyg.2018.00118 (PMC5811666; doi:10.3389/fpsyg.2018.00118)

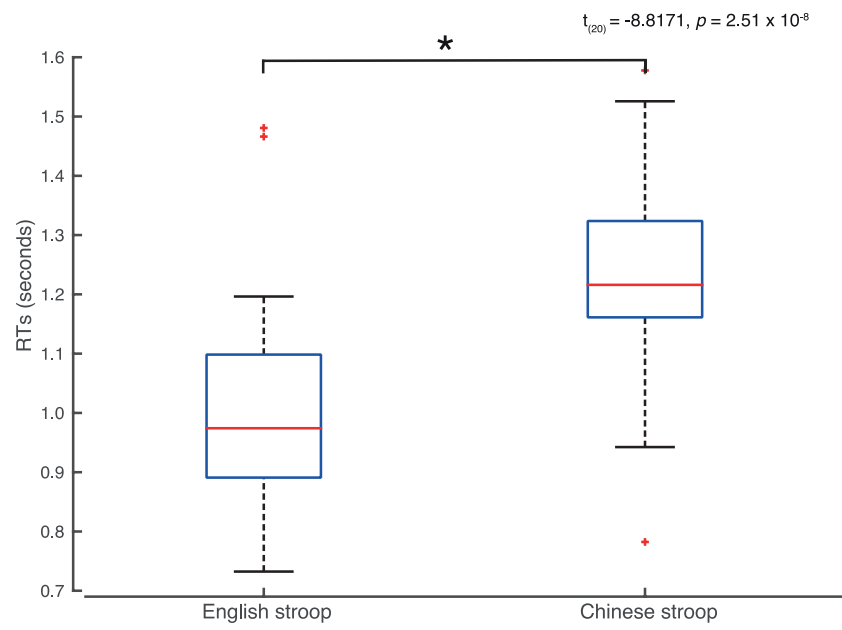

**Figure S6.** Statistical difference in students' RTs between English and Chinese Stroop.

Supplement: Supplementary file 6 [file Image_6.pdf]
